# Supplementary material for: Regulation of Plasmodesmata Function Through Lipid-Mediated PDLP7 or PDLP5 Strategies in Arabidopsis Leaf Cells
Source: Plants (Basel). 2026 Jan 4;15(1):145. doi: 10.3390/plants15010145 (PMC12787549; doi:10.3390/plants15010145)
Supplement: Supplementary file 1 [file plants-15-00145-s001.zip › plants-4033706-supplementary.pdf]

Supplementary Figure S1

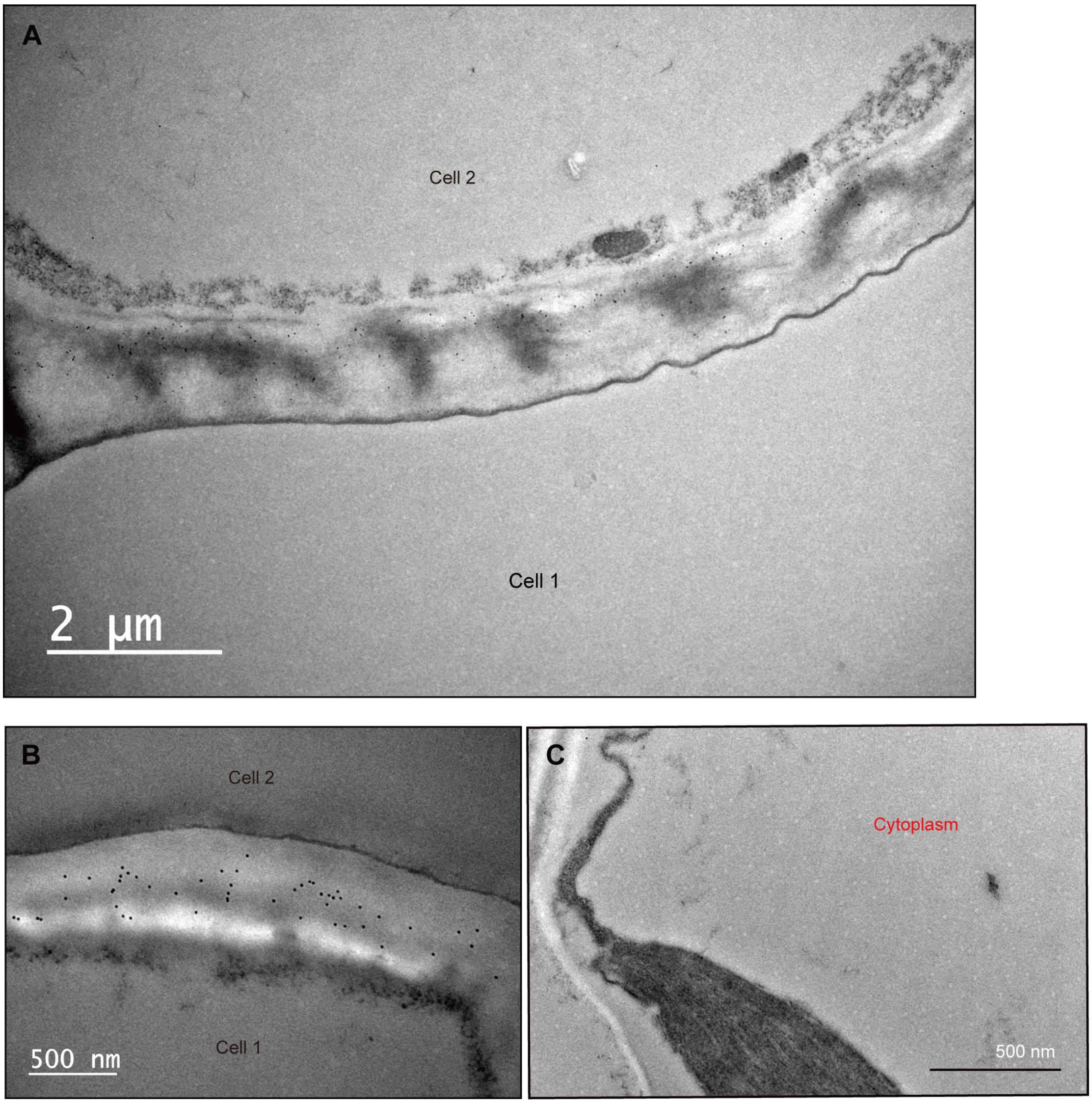

Supplementary Figure S1 | Immunoelectron TEM observation anti PDLP7.

(A-B) The enrichment of 10 nm gold particles indicates that PDLP7 is localized at the plasmodesmata and PM, rather than in cytoplasmic organelles such as chloroplasts (C).

Supplementary Figure S2

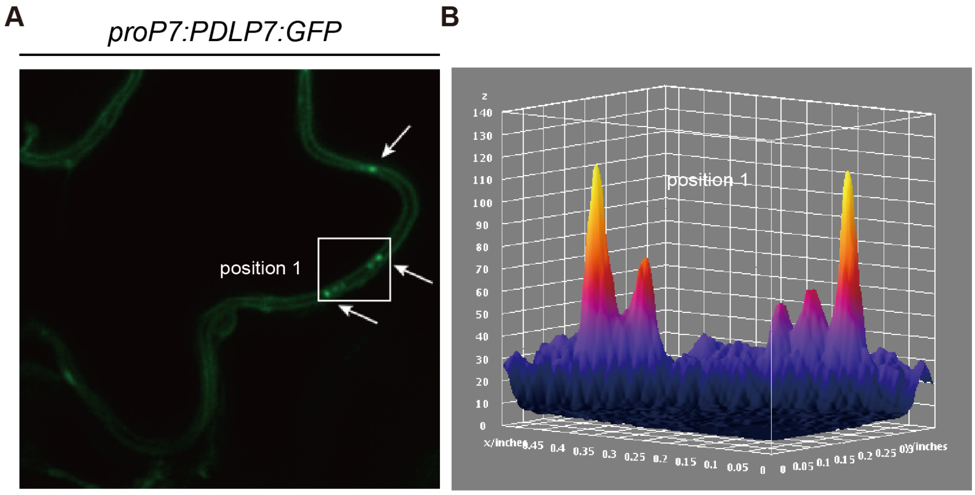

Supplementary Figure S2 | Observation of PDLP7:GFP driven by its native promoter.

(A) Punctate fluorescence produced by *proP7:PDLP7:GFP* is specifically localized between the plasma membranes (white arrows), as confirmed by confocal microscopy. (B) The protein distribution surface plot was generated from positions 1 in supplementary figure 2A.

Supplementary Figure S3

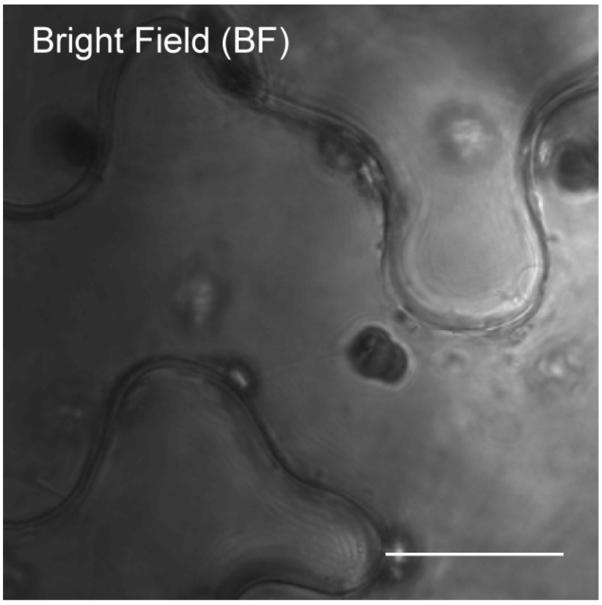

Supplementary Figure S3 | Bright field image of PDLP7:RFP with callose in Figure 1G. Scale bar, 20 μm.

Supplementary Figure S4

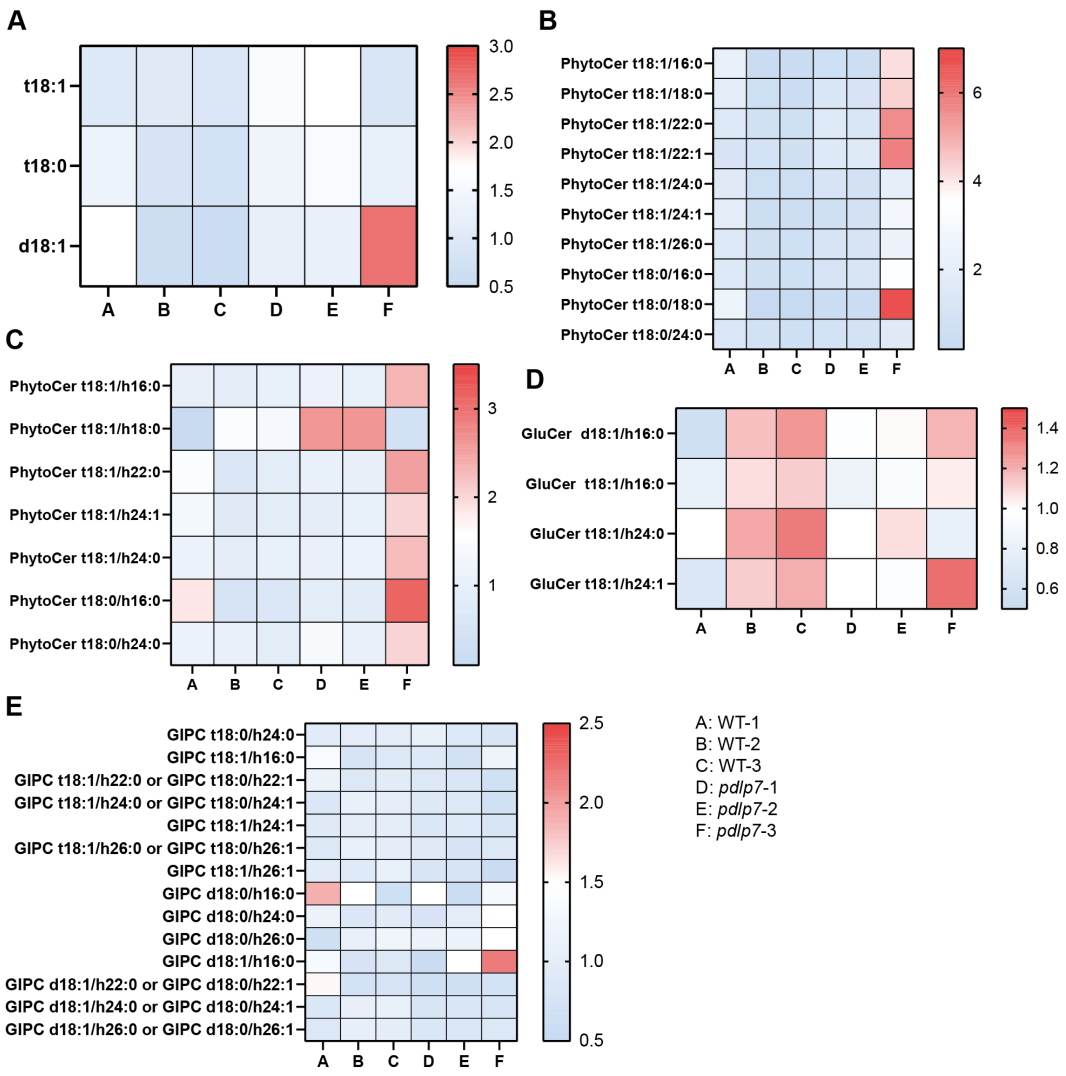

Supplementary Figure S4| Sphingolipidomic analysis of PD Components in wild-type and *pdlp7* mutant plants.

A)-(E) Heat map for the level of LCBs (A) , Cers (B), hyCers (C), GlcCers (D) and GIPCs (E) species. The average data of sphingolipid content for each species were normalized using a log2 calculation method. Values are means (n = 4).

Supplementary Figure S5

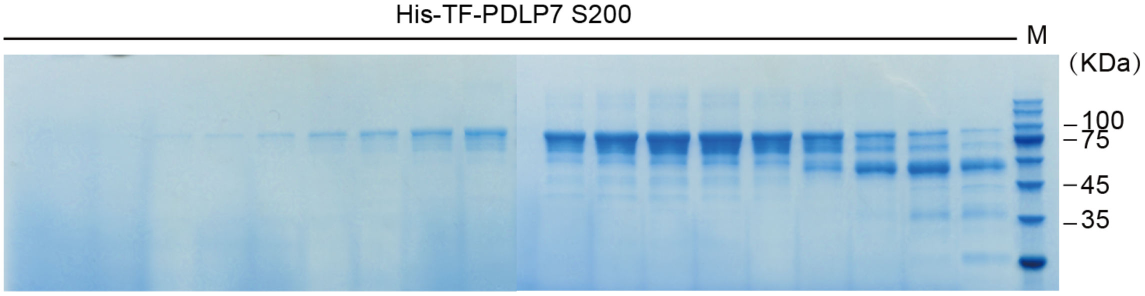

Supplementary Figure S5 | Purification of His-TF-PDLP7 proteins by Superdex 200 size exclusion chromatography.

Coomassie blue staining of purified His-TF-PDLP7 protein (~81 KDa). According to the elution time (from left to right), the proteins collected from the Superdex 200 chromatogram (500 μL per tube) were analyzed by SDS-PAGE.

Supplementary Figure S6

Sample：	1. Thyroglobulin (Mr 669 000)
	2. Ferritin (Mr 440 000)
	3. Aldolase (Mr 158 000)
	4. Conalbumin (Mr 75 000)
	5. Ovalbumin (Mr 44 000)
	6. Carbonic anhydrase (Mr 29 000)
	7. Ribonuclease A (Mr 13 700)

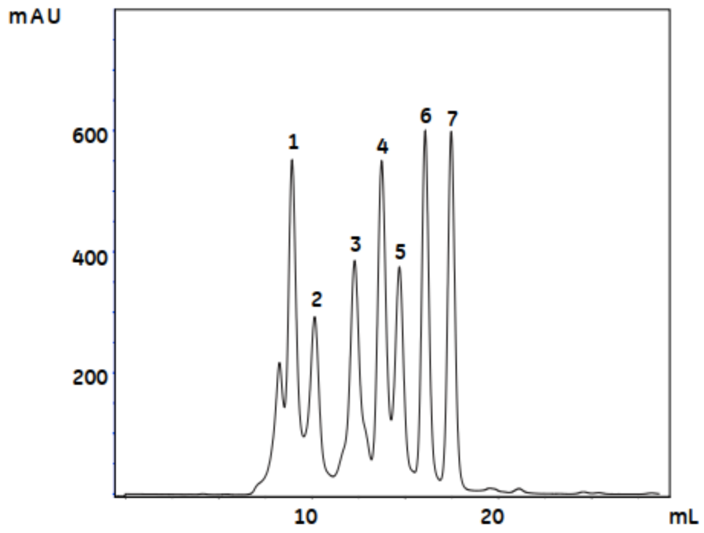

Supplementary Figure S6 | Typical chromatogram from a function test of Superdex 200.

The numbers above the chromatograms indicate the elution volumes of protein standards with different molecular weights (Da). This figure is used to determine the MW of the purified His-TF-PDLP7 protein, as referenced in the Instructions for Use provided by Cytiva (https://www.cytivalifesciences.com/en/us/shop/chromatography/prepacked-columns/size-exclusion/superdex-200-increase-small-scale-size-exclusion-chromatography-columns-p-06190).

Supplementary Figure S7

Protein FDR Confidence:	Master	Accession	Sum PEP Score	Coverage [%]	Peptides	Unique Peptides
High	Master Protein	AT1G70690.1	1.68	5	1	1
High	Master Protein	AT1G70690.1	6.8	5	1	1
High	Master Protein	AT1G70690.1	24.093	15	3	3

Supplementary Figure S7 | PDLP7 can co-immunoprecipitate the PDLP5 protein *in vivo*.

In three independent co-immunoprecipitation (co-IP) experiments, PDLP5 (AT1G70690.1) was detectable in the mass spectrometry datasets. Notably, in one of these experiments, both the total number of peptides and the number of unique peptides identified for PDLP5 were three ($\geq$2 is considered as reliable protein).

Supplementary Figure S8

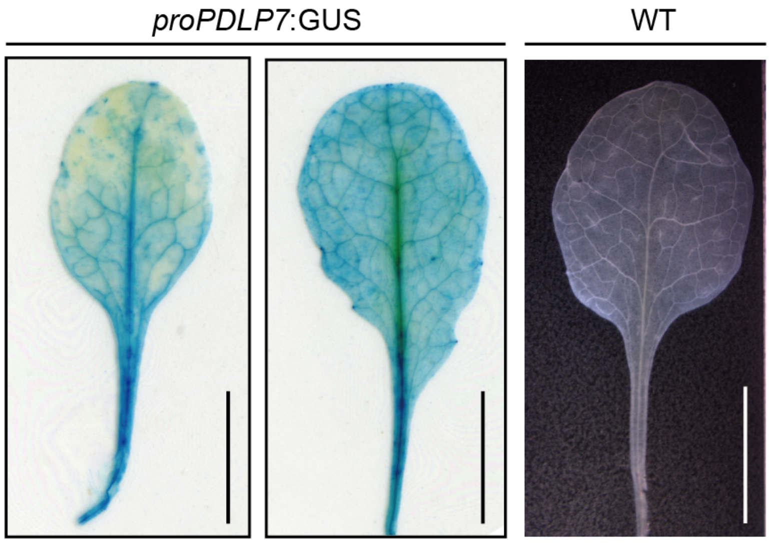

Supplementary Figure S8 | GUS staining of *proPDLP7:GUS Arabidopsis* transgenic lines.

The GUS staining indicate that PDLP7 is primarily localized in vascular tissues such as leaf veins and petioles. Scale bars, 5 mm.

Supplementary Figure S9

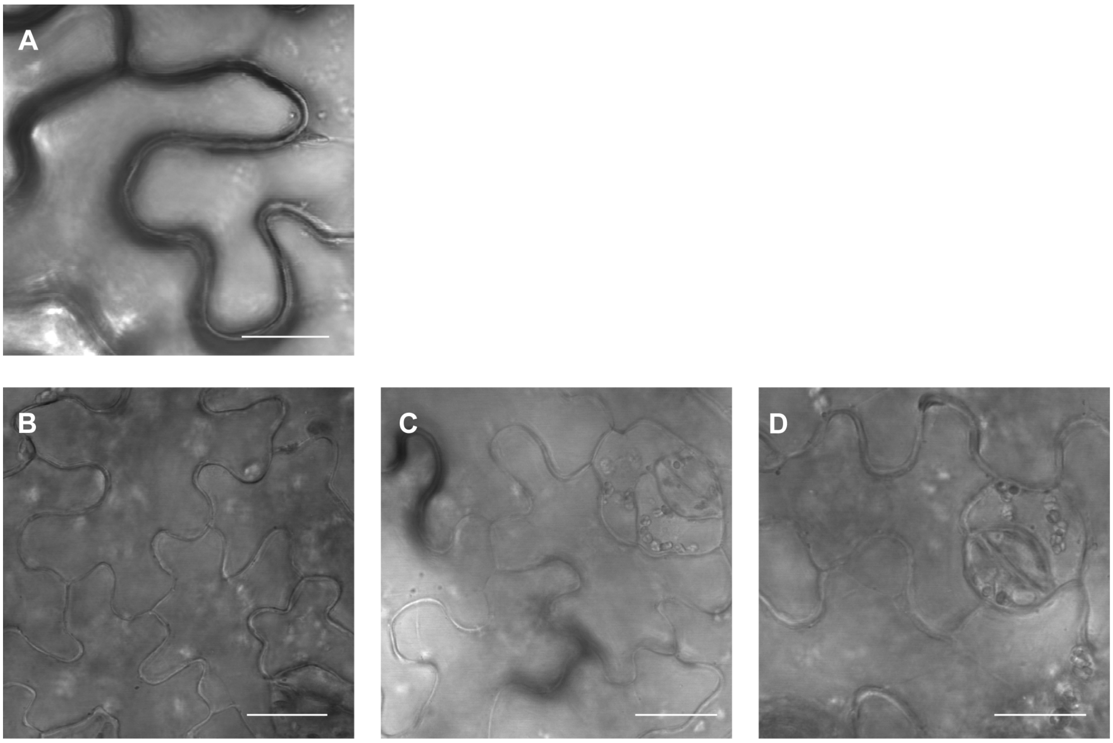

Supplementary Figure S 9 | Bright field images of PDLP7:GFP with PDLP5:mcherry (A) in Figure 4B and BiFC images in Figure 4D (B-D) respectively. Scale bar, 20 μm.

Supplementary Figure S10

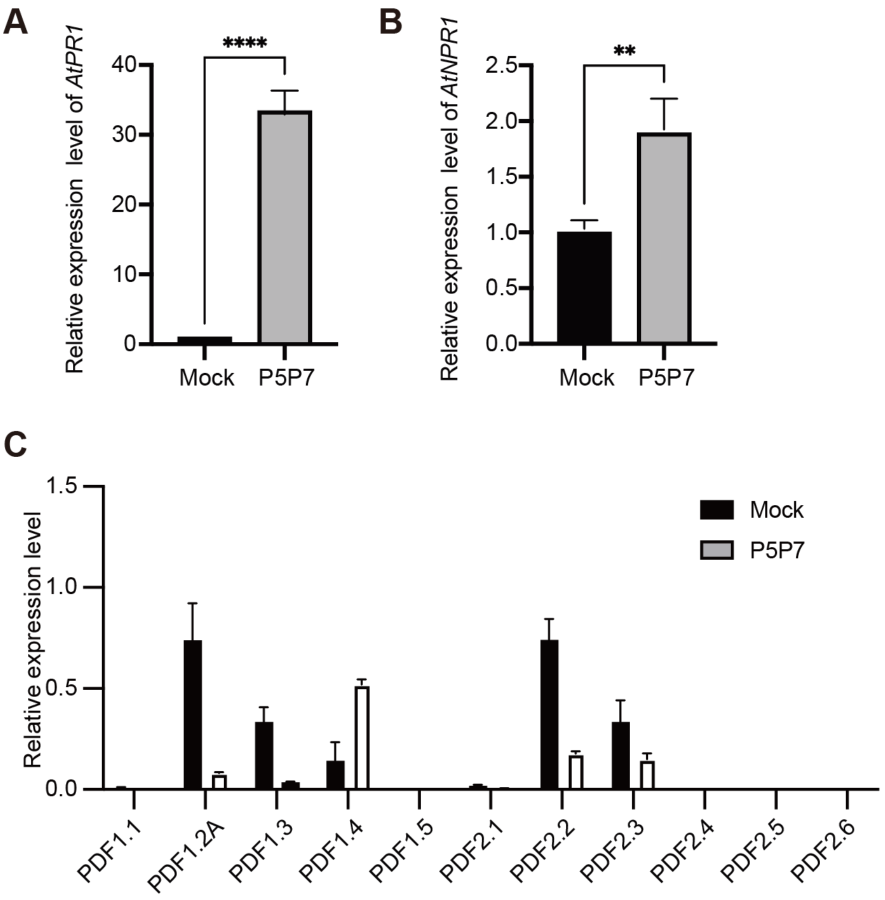

Supplementary Figure S10 | Relative expression level of *Arabidopsis PR1*, *NPR1* and *PDF*s.

(A) In *Arabidopsis* materials co-overexpressing PDLP5 and PDLP7 (P5P7), the transcriptional levels of *PR1* and *NPR1* were significantly elevated compared to Mock plants, with *PR1* demonstrating the most pronounced increase. (B) In contrast, *PDF*s did not exhibit a notable upward trend. Statistical significance was determined using the Student's t test. ***P* < 0.01, *****P* < 0.0001.
